# Reconstruction option of abdominal wounds with large tissue defects

**DOI:** 10.1186/1471-2482-14-50

**Published:** 2014-08-08

**Authors:** Martin Hutan, Christian Bartko, Ivan Majesky, Augustin Prochotsky, Jaroslav Sekac, Jan Skultety

**Affiliations:** 1 II.nd Surgical Clinic of Medical faculty Comenius University, University Hospital Bratislava, Hospital of st. Cyril and Methodius, Antolska 11, Bratislava 85107, Slovakia

**Keywords:** NPWT, Split skin grafting, Abdominal wall defect

## Abstract

**Background:**

Abdominal wall defects result from trauma, abdominal wall tumors, necrotizing infections or complications of previous abdominal surgeries. Apart from cosmetics, abdominal wall defects have strong negative functional impact on the patients.

Many different techniques exist for abdominal wall repair. Most problematic and troublesome are defects, where major part of abdominal wall had to be resected and tissue for transfer or reconstruction is absent.

**Case presentation:**

Authors of the article present operative technique, in which reconstruction of abdominal wall was managed by composite polypropylene mesh with absorbable collagen film, creation of granulation tissue with use of NPWT (negative pressure wound therapy), and subsequent split skin grafting.

Three patients with massive abdominal wall defect were successfully managed and abdominal wall reconstruction was performed by mentioned technique. Functional and cosmetic effect is acceptable and patients have good postoperative quality of life.

**Conclusions:**

Patients with giant abdominal defects can benefit from described technique. It serves as the only option, with which abdominal wall is fully reconstructed without need for the secondary intervention.

## Background

Abdominal wall defects result from trauma, abdominal wall tumors, necrotizing infections, or complications of previous abdominal surgeries. Apart from cosmetic point of view, abdominal wall defects have strong functional impact on the patients’ quality of life, since abdominal wall as a functional component of generation of Valsalva maneuver assists coughing, urination and defecation.

Many different techniques exist for abdominal wall repair. The mostly used are component separation technique [1], with support of absorbable/non-absorbable mesh [2], use of bioprosthetic materials [3], acellular dermal matrix, and use of local or distal flaps [4].

Giant abdominal wall defects with absent tissue for reconstruction pose a great challenge for the surgeon. One of the oldest techniques described (1953) is coverage of a defect with a pedicled musculofascial flap from the opposite side’s m. obliquus externus and its aponeurosis [5]. Another possibility of using autologous material is creation and utilization of the patient’s own skin as a corium transplant, which is immediately used with advantages of low infection rate and excellent acceptance of the host [6]. Use of absorbable meshes found its place in a temporary abdominal wall support in contaminated conditions, enhancing the likelihood of subsequent successful placement of a permanent mesh [7]. Technique may also be used with success as coverage after creation of laparostoma under the auspices of a planned hernia repair [8]. In recent years, the use of biological meshes with their low risk for graft rejection, complications, and infection, as compared to nonabsorbable ones, represent an innovation for hernia repair and abdominal wall reconstruction. However, elevated costs suggest evaluation of its use on case-by-case basis [9]. Use of tissue expanders is to this time reserved mainly for the pediatric area, where they are used for reconstructions of complex abdominal wall defects, usually resulting from omphalocele [10].

Most readily in such cases, use of nonabsorbable prosthetic material may be utilized, but with its limits in mind. These are predilection towards infection, creation of biofilm, and reluctance for granulation tissue formation if left open [11].

The incidence of infection in ventral hernia repair is as high as 8% [12,13], while the incidence of postoperative mesh infection is 1-2% [14]. The development of infection was shown to be dependent on two aspects: mesh type and surgical technique. Polypropylene meshes showed lower infection rates (2-4,2%) as opposed to ePTFE meshes (up to 9,2%) [15,16]. If we cannot provide primary closure of the wound, the incidence of infection will multiply.

Negative pressure wound therapy (NPWT) gained its place in the treatment of complicated wounds including those with massive infectious burden. It provides a closed moist environment with removal of excess fluid and promotion of granulation tissue formation even on bradytrophic surfaces, such as prosthetic implants, where spontaneous overgranulation is very slow and troublesome. The use of NPWT can be found in the prevention of complications in open incisonal hernia repair [17], in the treatment and salvage of infected meshes [18-20], and in the granulation tissue formation over bradytrophic tissues [21].

Among large number of treated patients arise ones, which suffer from such massive tissue loss that none of the surgical methods is appropriate. The use of prosthetic material is the only option in these defects, providing durable, functional and lasting repair. This option, however, comes along with a susceptibility to bacterial colonization and biofilm formation on the prosthetic material. If there is no viable tissue present for coverage, the overgrowth of the graft is destined to failure. NPWT could provide temporary coverage of the wound as well as time and conditions needed for recovery and healing. In such cases the use of NPWT may be the only option for salvage of abdominal wall and even the life of a patient.

The combination of both, i.e. the use of prosthetic material for reconstruction with the use of negative pressure, could provide solution for adverse aspects of both methods. On one hand prosthetic material provides strength and functionality of the abdominal wall, on the other NPWT indirectly prevents infection of the graft as well as enhances granulation tissue formation over the implant. Wound, which is prepared in such way, can be covered with split skin grafting. Up to this date, no such technique has been described in the literature. Authors’ hypothesis is that successful management of patients with massive abdominal wall defects may be accomplished with this technique.

## Case presentation

**Figure 1:**
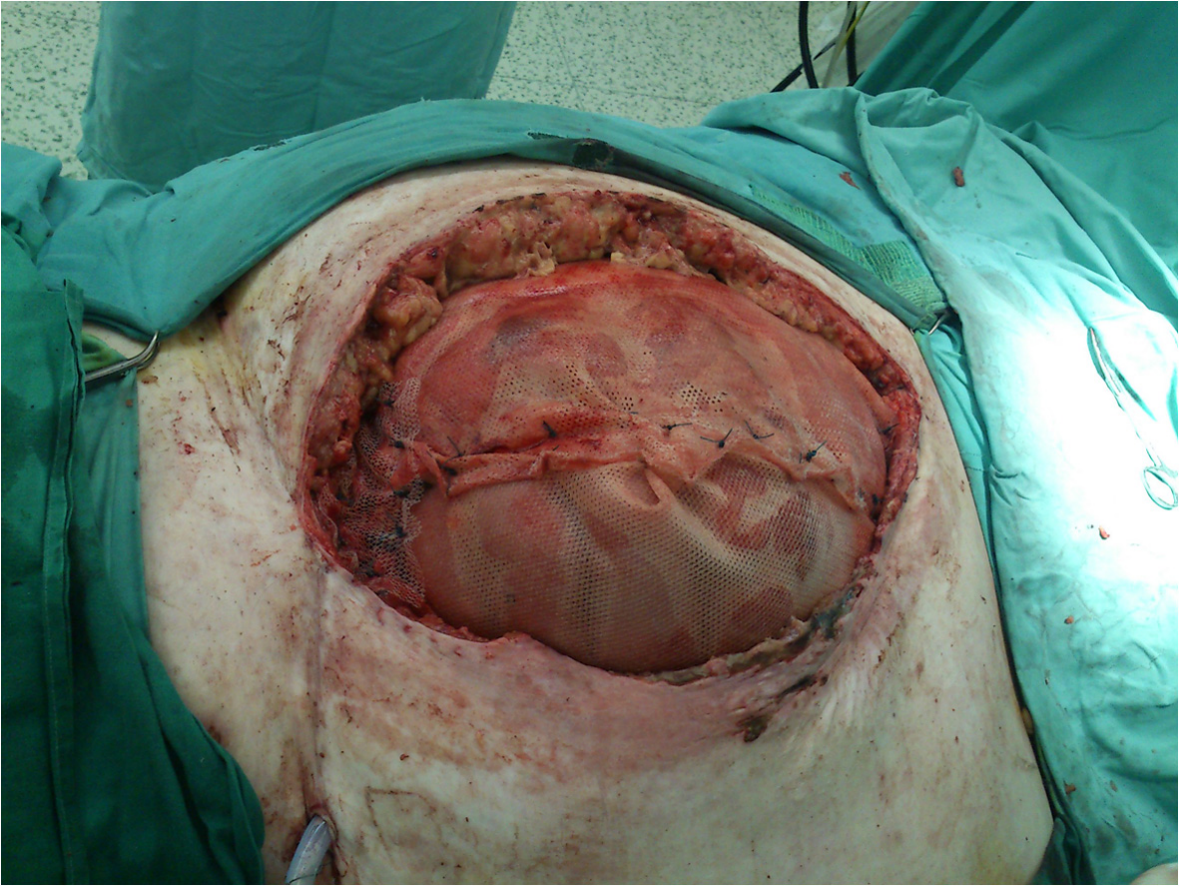
**Primary operation.** Patient 3, peroperative view with use of two 30 × 20 cm dual sided meshes.

**Figure 2:**
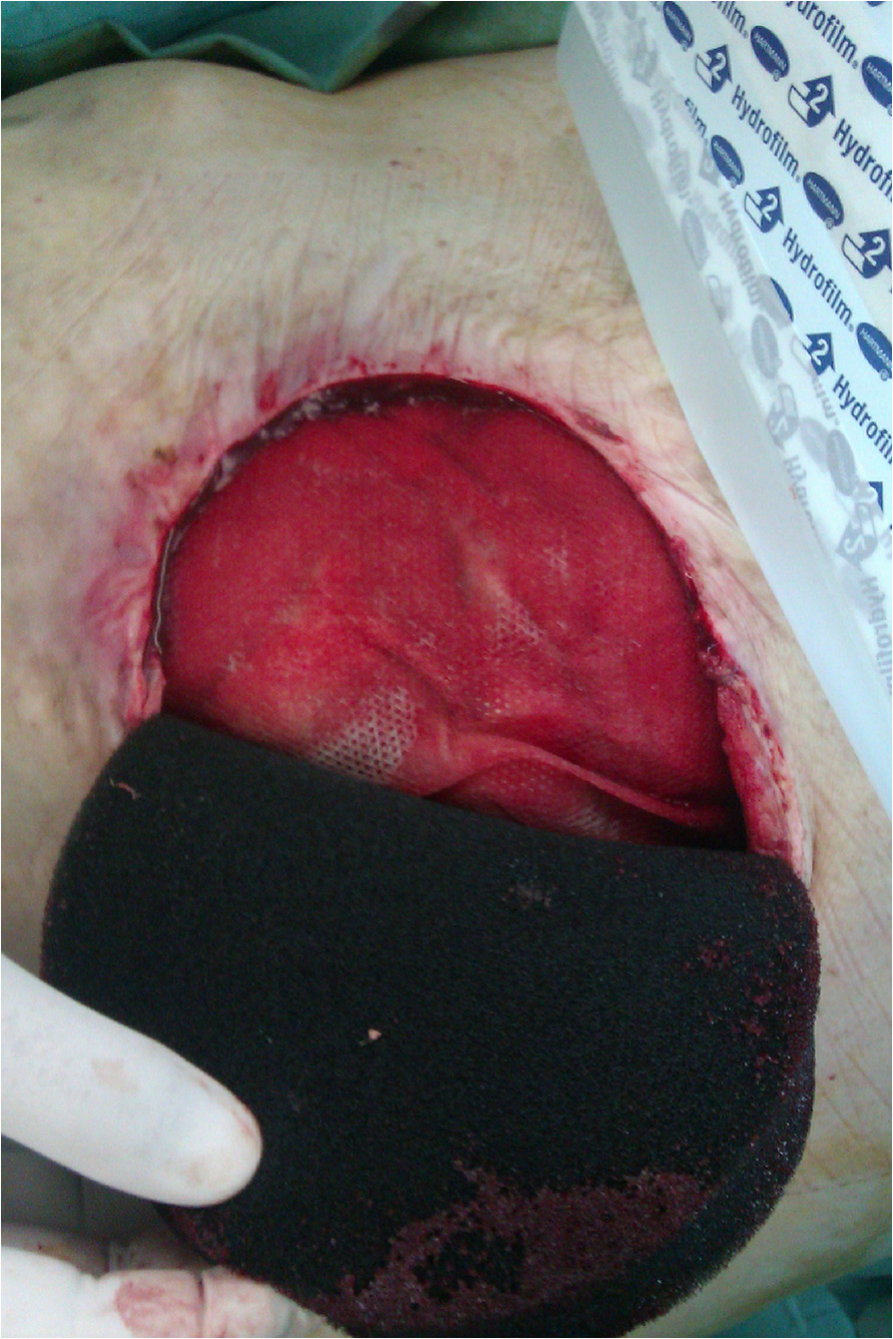
**Application and redress of NPWT.** Patient 2, progress of mesh overgranulation.

**Figure 3:**
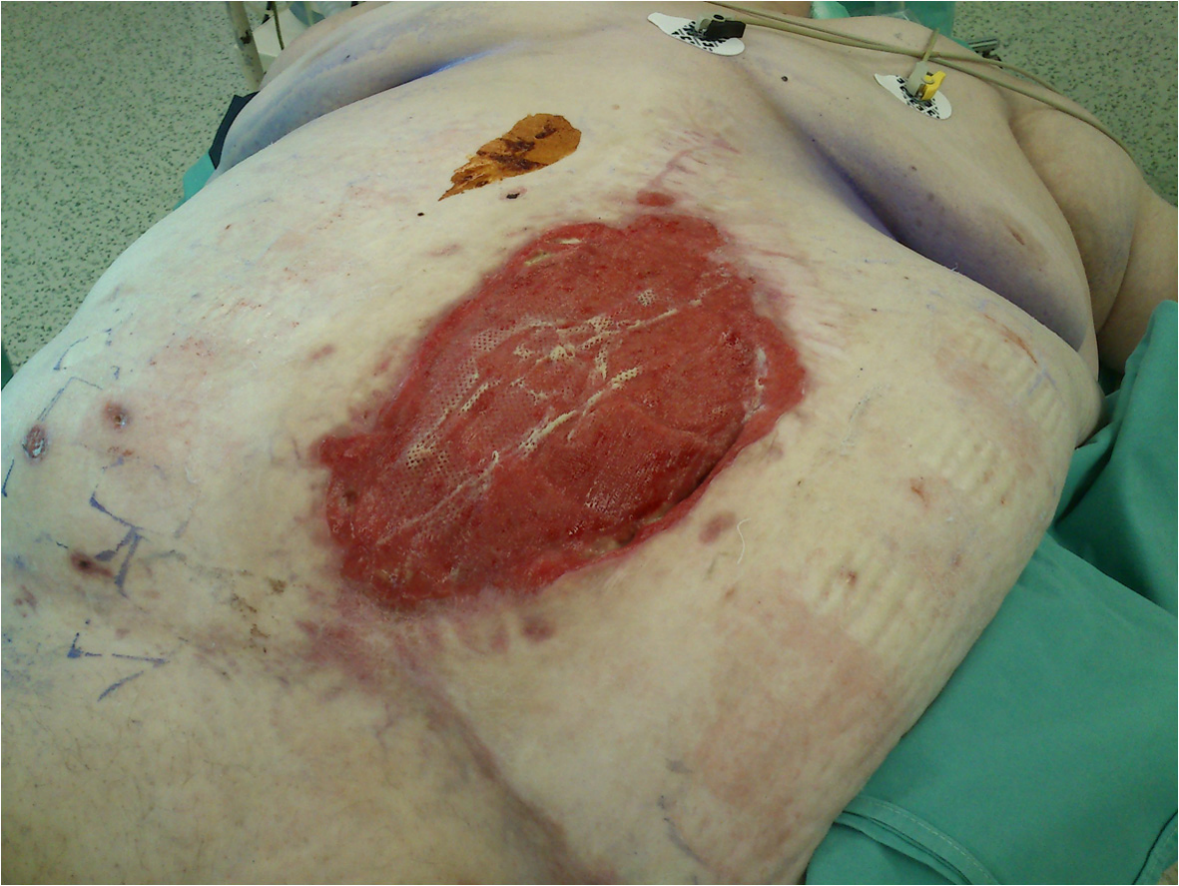
**Finalization of NPWT treatment.** Patient 3, overgranulated wound prepared for split skin grafting.

**Figure 4:**
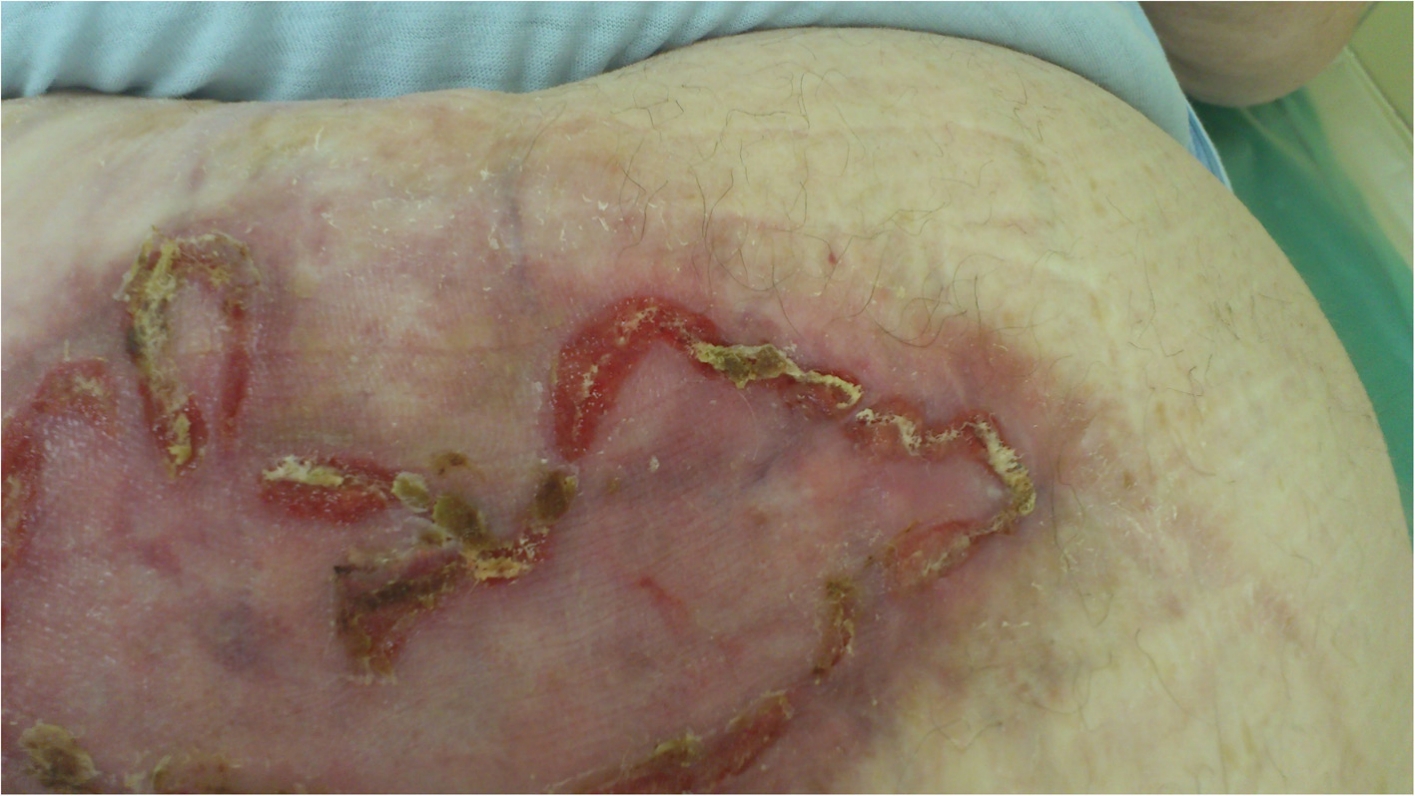
**Split skin grafting.** Patient 3, state after split skin grafting.

We present three patients, in whom the only possibility of abdominal wall reconstruction was the use of a polypropylene mesh with adhesion prevention film, enhancement of the growth of granulation tissue with use of NPWT, and subsequent split skin grafting. The mesh used in all three patients was Parietene Composite 3020 (Covidien, Dublin, Ireland), which is a composite polypropylene mesh with an absorbable collagen layer. This mesh is a standard double layer mesh, used in authors’ department for sublay hernia repairs and any other intraabdominal mesh placement techniques. After resection of necrotic (patient 1 and 3) or inadequate tissue (patient 2), we sutured the mesh under the fascia in sublay fashion using single non-resorbable sutures, overlapping the mesh by at least 3-4 cm under the edge of the fascia, and placing at least two rows of sutures with a distance of no more than 3 cm from the edge (Figure 1). We tried to interpose omentum between the mesh and the bowels, but complete coverage of intestinal loops was not possible in any of our patients. Immediately after the operation, NPWT was initiated by using continuous -125 mmHg during the first 48 hours. Then we switched to intermittent mode (-30 mmHg (0 mmHg in KCI ATS)/-125 mmHg, swap every 3 min). Black polyurethane (PU) foam was used in all three patients. We used KCI ATS (KCI, Texas, USA) in the first patient, the last two patients were treated with Vivano (PAUL HARTMANN AG, Germany). NPWT redress was done every 3 days (Figure 2). During redresses the wound was washed with only small amounts of physiological solution, no other agents were used. We decided to end the NPWT treatment when the wound bed was clean and filled with granulations, without clinical signs of critical colonization or infection, and no change of granulation growth was seen since the last redress (Figure 3). Split skin grafting was done at the time of termination of NPWT, donor skin was taken from the thigh using pneumatic dermatome set to the middle skin thickness. Wound was covered with vaseline gauze (Grassolind, Atrauman, PAUL HARTMANN AG, Germany) and redress was done on the fifth day. Outpatient follow up (Figure 4) was one year after hospital release for the first patient, the other two patients were followed for two years. Further follow up was taken up by their family practitioner. Quality of life was assessed verbally by patient responses to questions concerning mobility, self-care, adjustment to everyday work, need for change of their work or even retirement, need for pain medication, and overall subjective perception of the quality of life.

### Case 1

A 58-years-old man with a height of 1,78 m and a weight of 185 kg, suffered from a large ventral abdominal hernia. He was admitted for elective Pitanquy operation with hernia repair, which was done together with Lichtenstein hernioplasty. Patient suffered postoperatively from respiratory insufficiency and surgical site infection with necrotizing fasciitis. He plunged into massive sepsis with multiple organ dysfunction syndrome. With necessary necrectomy the patient ended with the defect in the abdominal wall sized 55 cm × 18 cm. Together with systemic intensive organ support we provided the defect with two Parietene 3020 dual sided meshes (Covidien, Dublin, Ireland) (sized 30 × 20 cm) sublay laterally to the lumbar region. We applied negative pressure wound therapy on the mesh (VAC ATS, KCI, Texas). Patient was hospitalized in anesthesiology department, with intensive organ support, artificial ventilation, and circulatory support with noradrenalin. Antibiotic therapy with combination of cephalosporins, quinolones, metronidazole was administered, at the time of initiation of NPWT changed to carbapenems. Meropenem was discontinued on 12^th^ day after initiation of NPWT. 4 weeks after initiation of treatment the patient underwent split skin grafting. During this time patient recovered from sepsis and MODS and was released from the hospital. He had no wound complications in the outpatient setting and no need for further surgery arisen. His quality of life is characterized by good mobility and self-care, appropriate adjustment for everyday work, and occasional use of painkillers.

### Case 2

A 50-years-old man was admitted for recurrent ventral incicatrical hernias. Patient was after four previous operations for ventral hernias. The perioperative finding was a massive, multilocular hernia from iliacal cristae to xiphoid with nearly whole area consisting of hernia sacs. Resecting this hernia sacks we created a defect sized 26 cm × 25 cm with practically no material for reconstruction. After component separation we used a sublay Parietene 3020 dual sided mesh (Covidien, Dublin, Ireland) over which we applied NPWT (Vivano, PAUL HARTMANN AG, Germany). 2^nd^ generation cephalosporin was administered as a prophylactic treatment; this was discontinued on day 8 after initialization of the therapy. After 8 dressing changes we ended NPWT treatment and applied split skin graft. The wound healed without complications and the patient has a good quality of life (good self-care, perfect mobility, occasional use of painkillers).

### Case 3

A 62-years-old woman with a height of 160 cm and a weight of 164 kg was admitted because of suspected tumor of the gallbladder. The operation was performed through the previous hernia sacs in medial laparotomy. In postoperative course the dehiscence of the wound occurred. This happened with the loss of domain formation after necrotization of the edges of the abdominal wall. After necrectomy a defect of approximately 45 cm × 30 cm opened. For reconstruction we used two pieces of sublay dual sided mesh Parietene 3020 (Covidien, Dublin, Ireland) and supported granulation tissue formation with the use of NPWT (Vivano, PAUL HARTMANN AG, Germany). Because of surgical site infection 2^nd^ generation cephalosporin together with metronidazole was initiated one day before the operation for wound dehiscence, and was discontinued on day 5 of NPWT therapy. After twelve dressing changes sufficient granulation tissue was built up. Afterwards, we applied a split skin graft on the granulated wound bed. Patient was released from the hospital, was feeling well, and up to this date, there were no complications. She has good self-care and mobility with occasional use of painkillers.

Besides the abdominal wall reconstruction all of the patients were successfully managed for their underlying disease. Comorbidities, medications used, and duration of the hospitalization are stated in Table 1. Functional and cosmetic effects are acceptable and patients have good postoperative quality of life.

**Table 1:** List of patients

	**Patient 1**	**Patient 2**	**Patient 3**
Comorbidities	-Obesity (BMI = 58,38)	-Arterial Hypertension	-Obesity (BMI = 64,06)
	-Arterial Hypertension	-Ischemic Heart Disease	-Ischemic Heart Disease
	-Ischemic Heart Disease	-Operations: cataracta l.dx., umbilical hernia (1996), recurrence and reoperation with mesh (1999, 2000, 2007), 2009 together with fistula to mesh extraction	-Arterial Hypertension
	-Diabetes type II on Insulin		-Diabetes type II on Insulin
	-Hyperlipidemia		-Hyperlipidemia
			-Operations: Hernioplasty for epigastric hernia (2000)
Medications used	-Chronic medication (antihypertensives, betablocker, insulin, hypolipidemics)		-Chronic medication (antihypertensives, insulin, hypolipidemics, aspirin)
	-In-hospital medication: infusion therapy, all in one, albumin, LMWH, PPI, antibiotics (betalactams, carbapenems, chinolones, metronidazole, fluconazole), insulin, adrenalin, noradrenalin, furosemide, ambroxol, analgetics, sufentanil, midazolam	-Chronic medication (antihypertensives)	-In-hospital medication: infusion therapy, LMWH, PPI, antibiotics (betalactams), ambroxol, analgetics, insulin
		-In-hospital medication: infusion therapy, LMWH, PPI, antibiotics (betalactams), ambroxol, analgetics	
Duration of the hospitalization	65 days	51 days	66 days

Patients, comorbidities, medications used and time of hospitalization.

## Discussion

The most problematic and troublesome issues in abdominal wall repair are defects, in which the major part of the abdominal wall had to be resected and no sufficient tissue is left for the closure of abdomen.

The aim of the reconstruction should be the coverage of a wound as a prevention of infectious complications, and reconstruction of the abdominal wall, that will ensure its functionality.

Use of component separation technique in these patients might be insufficient, and even with wide incisions in aponeurosis of m. obliquus externus or in sheath of mm. recti abdominis the extension of the abdominal wall will not sufficiently cover the defect. Nevertheless it is a method, with which we can reduce the size of a defect and use for preparation for method presented [1]. Use of corium transplant is a safe method, but very rarely applicable since it requires both experienced surgeon and plastic surgeon skilled in such technique available in operating theatre. This method is also being timely and technically highly requiring [6]. As previously stated, use of absorbable mesh has its place usually as a temporary abdominal wall support and even though indicated for use in contaminated conditions and enhancing the likelihood of subsequent successful placement of a permanent mesh, secondary intervention is necessary for full functionality of abdominal wall [7]. Use of non-absorbable mesh in septic conditions with no coverage has almost no chance for infection-free and complication-free ingrowth [12-16]. In any of these techniques we are still dealing with a wound, in which even after complete reconstruction of abdominal wall (if possible), skin coverage is missing and the wound has need for alternative temporary coverage.

Use of pedicled flaps transfer would be a good choice if it wouldn’t pose such extensive operative intervention for such defects. Patients in critical conditions are in need of as quick and effective procedure as possible.

NPWT as a wound healing method can be considered as a system of a wound coverage, which is as close as possible to the ideal wound coverage. It provides a closed, evenly moist environment, protected from the intrusion of the bacteria, with effective elimination of excessive exudation.

Similar techniques, where NPWT serves as a temporary abdominal closure with defect fascia are well known. One of the first described and used was “Vacuum pack” described by Brock and Barker [22,23]. The same author in 2007 describes use of negative pressure with use of polyurethane foam in vacuum assisted fascia closure [24]. The latter described techniques are aiming at restoration of the fascia integrity in reconstruction of the defect. The big step to this was use of static compression sutures described by Navsaria [25]. Retraction of the fascia is, however, better provided by dynamic sutures or even with use of mesh mediated traction to fascia [26,27]. These methods are generally used in patient with open abdomen. Treatment options in certain complications of open abdomen, such as “loss of domain formation” by retraction of abdominal wall in prolonged open abdomen, pose very similar challenge, and these techniques led us to the presented method, that we use in different indication while facing the same problem.

If no abdominal wall reconstruction is possible, the simplest method is application of abdominal set of NPWT. After creation of granulation tissue, split skin grafting can be utilized. Drawback of such method is an inevitable complication – massive ventral hernia will form in the scar after maturation of the collagen. Such technique provides no functional or esthetic effect, and leads to low quality of life together with morbidity resulting from loss of functional abdominal wall. Secondary hernia operation in such condition would pose a great challenge for non-complicated recovery, having high risk of perioperative complications in terms of creation of enterocutaneous/enteroatmospheric fistula as a consequence of adhesiolysis of the visceral block.

The method presented here creates a functional abdominal wall (with use of polypropylene implant) where infection of the mesh is substantially lowered by the use of NPWT. Using advantages of both methods the patient gets a unique chance for recovery and restoration of the abdominal wall in one hospitalization. Biggest advantages of such technique get patients, in which such condition is a result of infectious complication (such as necrotizing fasciitis). Utilizing wound coverage by NPWT (which also serves as a source control aspect of infection) the patient is able to recover from sepsis and MODS. Intensive systemic therapy and organ support therapy is an essential part of treatment in such patients, and without such any local wound treatment is inadequate.

This method can be considered as a salvage therapy of selected patients with complicated and complex abdominal wall defects. We used it in patients with critical state, sepsis and MODS. All of our patients had serious comorbidities (ischemic heart disease, high blood pressure), first and third patient was extremely obese and suffered from diabetes on insulin therapy. The drawback of such technique is its cost and need for long hospitalization. On the other side, in presented patients, this was the only option that solved the massive defect without need for the secondary operation.

## Conclusions

Giant abdominal wall defects pose a great challenge, particularly when the situation is complicated by the absence of usable tissue, the clinical state of the patient, and his comorbidities. This short case series demonstrated that the use of dual sided mesh, the support of granulation tissue formation with prevention of infection by NPWT, and subsequent split skin grafting is appropriate to manage large abdominal wall defects while ensuring both closure and functionality.

## Consent

Written informed consent was obtained from the patients for publication of these case reports and any accompanying images. A copy of the written consent is available for review by the Editor of this journal.

## Abbreviations

LMWH: Low molecular weight heparin; MODS: Multiple Organ dysfunction syndrome; NPWT: Negative pressure wound therapy; PPI: Proton pump inhibitors.

## Competing interests

First author (Martin Hutan, MD, PhD) is a member of Competence Network, international expert group for use of negative pressure wound therapy sponsored by PAUL HARTMANN AG, Germany. No organization is financing this manuscript, no financial reimbursement for this article will be obtained and no other competing financial interests are present. Other authors have no competing interests.

## Author’s information

Martin Hutan, MD, PhD – born in 1981, general surgeon with interest in antibiotic therapy (investigator in the CIAO study) and wound therapy, co-founder and board member of Slovak Wound Healing Society, member of Competence Network, international expert group for NPWT, editorial board member of Negative Pressure Wound Therapy journal (Research Publisher, CA, USA). Author or coauthor of 97 publications, first author of 46 presentations in Slovak and international congresses, coauthor of a book on NPWT published in Czech Republic (Negative Pressure Wound Therapy, Maxdorf, Prague), coauthor of textbook of surgery in Slovak Republic (Principles of general surgery and its specialties, Comenius University, Bratislava).

## Authors’ contributions

MH managed NPWT in all patients, carried on split skin grafting of all patients, was operating surgeon of the first patient, developed the presented method, drafted the manuscript. CB revised the article and contributed with the information support. IM was operating surgeon of the two last patients, contributed to development of the method. AP revised the article and contributed with the information support. JS revised the article and contributed with the information support. JS is the chief of the department, overviewed the management of the patients, contributed with the information support. All authors read and approved the final manuscript.

## Pre-publication history

The pre-publication history for this paper can be accessed here:

http://www.biomedcentral.com/1471-2482/14/50/prepub
